# The power of synthetic biology for bioproduction, remediation and pollution control: The UN's Sustainable Development Goals will inevitably require the application of molecular biology and biotechnology on a global scale

**DOI:** 10.15252/embr.201745658

**Published:** 2018-03-26

**Authors:** Víctor de Lorenzo, Kristala LJ Prather, Guo‐Qiang Chen, Elizabeth O'Day, Conrad von Kameke, Diego A Oyarzún, Leticia Hosta‐Rigau, Habiba Alsafar, Cong Cao, Weizhi Ji, Hideyuki Okano, Richard J Roberts, Mostafa Ronaghi, Karen Yeung, Feng Zhang, Sang Yup Lee

**Affiliations:** ^1^ Global Future Council on the Future of Biotechnologies World Economic Forum Geneva Switzerland; ^2^ Systems Biology Program National Center of Biotechnology CSIC Madrid Spain; ^3^ Department of Chemical Engineering MIT Cambridge MA USA; ^4^ Center for Synthetic and Systems Biology MOE Lab for Industrial Biocatalysis Tsinghua‐Peking University Center of Life Sciences School of Life Sciences Tsinghua University Beijing China; ^5^ Center for Synthetic and Systems Biology Tsinghua University Beijing China; ^6^ Olaris Therapeutics, Inc. Cambridge MA USA; ^7^ BioInnovators Europe Berlin Germany; ^8^ Department of Mathematics Imperial College London London UK; ^9^ Department of Micro‐ and Nanotechnology Center for Nanomedicine and Theranostics Technical University of Denmark Kongens Lyngby Denmark; ^10^ Khalifa University Center for Biotechnology Khalifa University Abu Dhabi United Arab Emirates; ^11^ University of Nottingham Ningbo China; ^12^ Kunming University of Science and Technology Kunming Yunnan China; ^13^ Department of Physiology Keio University School of Medicine Tokyo Japan; ^14^ New England Biolabs Ipswich MA USA; ^15^ Illumina Inc. San Diego CA USA; ^16^ The Centre for Technology, Ethics, Law, & Society, Law School King's College London London UK; ^17^ Broad Institute of MIT and Harvard Cambridge MA USA; ^18^ McGovern Institute for Brain Research at MIT Cambridge MA USA; ^19^ Department of Brain and Cognitive Sciences and Department of Biological Engineering Massachusetts Institute of Technology Cambridge MA USA; ^20^ Department of Chemical and Biomolecular Engineering (BK21 Plus Program) Korea Advanced Institute of Science and Technology (KAIST) Daejeon Korea; ^21^ The Novo Nordisk Foundation Center for Biosustainability Technical University of Denmark Lyngby Denmark

**Keywords:** S&S: Ecosystems & Environment, S&S: Technology, Synthetic Biology & Biotechnology

## Abstract

The UN's Sustainable Development Goals present a challenge for biotechnology to develop new environmentally‐friendly and sustainable products and production processes.

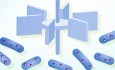

The agenda of the UN's *Sustainable Development Goals* (SDGs) 1 challenges the synthetic biology community—and the life sciences as a whole—to develop transformative technologies that help to protect, even expand our planet's habitability. While modern tools for genome editing already benefit applications in health and agriculture, sustainability also asks for a dramatic transformation of our use of natural resources. The challenge is not just to limit and, wherever possible revert emissions of pollutants and greenhouse gases, but also to replace environmentally costly processes based on fossil fuels with bio‐based sustainable alternatives. This task is not exclusively a scientific and technical one but will also require guidelines and regulations for the development and large‐scale deployment of this new type of bio‐based production. Some recent advances that can (or soon could) enable us to make progress in these areas—and several possible governance principles—need to be addressed.

### The potential of biotechnology

The transformative power of modern, science‐based biotechnology that started in the late 1970s has been accelerated by recent developments, such as massive DNA synthesis/sequencing, systems and synthetic biology, and CRISPR tools for genome editing. The interface of these disciplines and techniques with other flagship technologies of the ongoing Fourth Industrial Revolution 2, such as artificial intelligence, robotics, big data, ITs, and so on, will usher in a society, economy and industry that are very different from what we know today. So far, market forces have pushed most research efforts towards health‐related issues and agricultural productivity, as these areas can more easily harvest low‐hanging fruits of contemporary systems‐based biotechnology. But the spectacular advances in biomedicine and agricultural technologies are happening during an acute global environmental crisis caused by overpopulation, loss of biodiversity, greenhouse gas emissions and pollution. Thus, environmental sustainability is at the core of the *Sustainable Development Goals* (SDGs) proclaimed by the United Nations 1 in 2015. One key aspect of this pledge is the need to produce goods and products in a way that is both economically viable and ecologically sustainable. However, reducing and eventually stopping harmful emissions is not sufficient; it will also require large‐scale interventions to restore ecological balances and remove pollution by industrial and urban activities. Given this background, what can and should *Biotechnology 4.0*—biotechnology in the era of the Fourth Industrial Revolution—do; in particular, how could systems‐guided metabolic engineering and synthetic biology contribute to sustainability goals?

Given the ongoing climate change and the limits of fossil resources, bio‐based production of chemicals and materials from renewable resources is becoming increasingly important. While there are numerous possible applications of engineered biological systems, some of the most well‐known examples involve the rational design of microbial hosts for the production of various chemicals and materials. A good example is the construction of yeast strains for the production of the anti‐malarial drug artemisinin and opiates to provide a sustainable supply that is unaffected by environmental factors that could impact the plant source of these drugs. More recently, the integration of computational and experimental approaches has enabled the generation of a yeast strain that produces exceptionally high yields of lipids as biofuel precursors. Even non‐natural chemicals, such as gasoline and terephthalic acid, could be produced by microbial metabolic engineering 3.

### Evolution and design

Biology‐by‐design has also succeeded in producing biomaterials including polysaccharides, proteins, spider silk and diverse polyhydroxyalkanoates (PHAs). For example, microorganisms have been successfully engineered to efficiently produce PHAs, a family of diverse biopolyesters, for environmentally friendly packaging, medicine and smart materials (Fig 1). Beyond production of PHAs, metabolic engineers have developed microbial strains capable of producing even non‐natural materials such as poly(lactate‐co‐glycolate), an FDA‐approved biomedical polymer.

**Figure 1:**
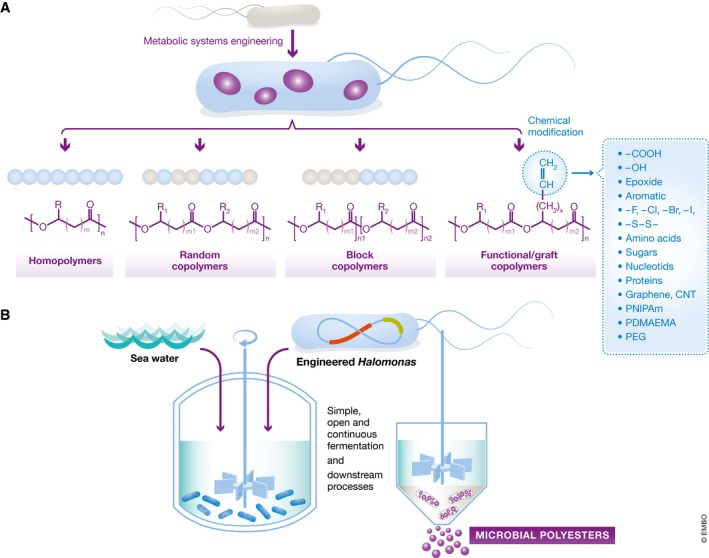
Bioplastic production (A) Polyhydroxyalkanoates (PHAs) and other polymers with diverse structures in the forms of homopolymers, random copolymers and block copolymers can be produced by microorganisms developed by systems metabolic engineering. These polymers can be further chemically modified to make functional and graft copolymers. (B) Industrial biotechnology based on extremophilic microorganisms (e.g., salt‐loving *Halomonas* bacteria) could significantly reduce the manufacture cost of PHAs and other bioproducts.

Processes and protocols for cultivating industrial microorganisms in a bioreactor are already well established but could be further improved by exploring the physicochemical properties of natural organisms, notably extremophilic bacteria. For example, a salt‐loving *Halomonas* strain that can grow under high osmotic pressure and high pH was recently engineered to produce chemicals, biofuels and other valuable compounds (Fig 1). It is now used to produce PHA under unsterilized conditions using seawater in open reactors made of ceramic, cement or plastic, which helps to save energy, fresh water and substrates. Similarly, environmental bacteria that are able to thrive in industrial sites with heavy chemical pollution became a treasure trove for research to find both robust chassis, such as *Pseudomonas putida*, and catalytic activities that can be used by the chemical industry 4.

One could then imagine developing a set of designer microbes that can be deployed as required to increase the range of compounds that can be produced biologically to meet human needs—from medicines to materials—or to remediate contaminants in the environment. Increasing the range of biological organisms that can be predictably and reliably engineered along with the scale of those engineering designs is a critical next step towards fully realizing the potential of bioproduction 5.

### *In silico* tools for design

Several important developments are still needed. These include an ability to rapidly engineer existing organisms already endowed with desirable phenotypic traits—for instance, a higher tolerance for organic solvents—as such complex phenotypes are often difficult‐to‐impossible to transfer to more tractable hosts. Additionally, understanding context dependence, such that engineered genetic circuits can be readily transferred to new biological frames while performing up to the original specifications, is essential to increase the speed of developing new workable chassis. Advances are also needed in computational biology such that the behaviour of engineered biological systems can be better predicted from the *in silico* design.

… sustainability also asks for a dramatic transformation of our use of natural resources.

Using mathematical modelling and computer simulations, designers can test system models for feasible system architectures, optimal combinations of parameters, or performance in regimes that would be otherwise infeasible or too costly to test *in vitro*. Mathematical models have been essential for computer‐aided design in diverse areas, including molecular dynamics simulations for drug design, constraint‐based models for metabolic engineering, and process models for bioreactor control. Mathematical models are also valuable tools to understand how gene circuits cause knock‐on effects on host physiology that could disrupt the performance of implanted devices. Model‐guided design will become increasingly important as synthetic biology creates larger systems that interface gene circuits with other cellular systems, such as signalling networks and/or metabolic networks. For example, dynamic metabolic engineering models have revealed how systems‐level properties, such as metabolic heterogeneity, can be controlled and even exploited for biosynthesis. Furthermore, new algorithms based on artificial intelligence and rapidly increasing big (bio)data will be needed to develop microbial cell factories and to optimize bioprocesses. Such advanced modelling and simulation can also help to meet the challenge of scaling‐up, speeding‐up and making large bioprocesses more predictable—the main obstacles for the still evident scepticism of the chemical industry for bio‐based manufacturing.

### Dealing with waste and pollution

Contemporary biotechnology has not only created new methods and processes to produce molecules and materials. It has also generated new approaches for managing, sensing and remediating pollutants, including the transformation of waste into value‐added molecules or energy. Unfortunately, for now, the commercial worth of environmental biotechnology is orders of magnitude lower than its biomedical and demand‐driven counterparts. Yet, the mounting evidence of climatic change caused by industrial CO_2_ emissions and other environmental calamities—water quality, plastic waste in marine ecosystems and loss of soluble phosphorus—increases the motivation to tackle these challenges (Fig 2).

**Figure 2:**
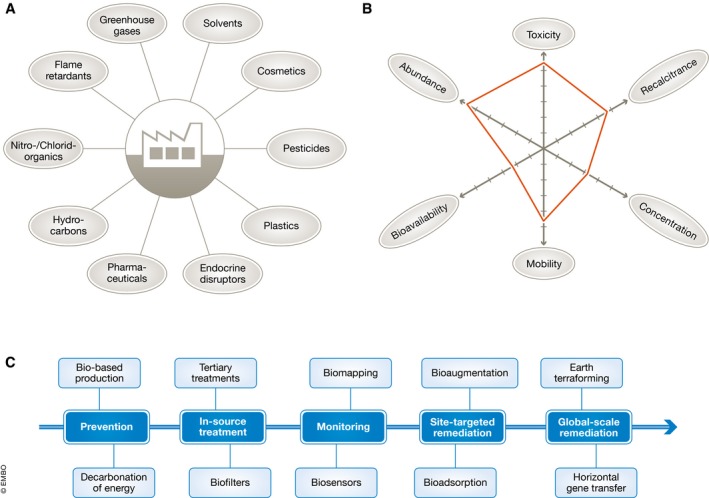
Advanced biotechnology for the environment Typical anthropogenic emissions and measures to manage them. (A) Industrial and urban activities generate molecules that impact negatively the functioning of the biosphere. (B) The profile of an environmental pollutant is defined by the six parameters indicated, the outcome of which frames the bioremediation strategy. (C) Bio‐based approaches to tackle environmental pollution, from prevention to global‐scale remediation. The direction of the arrow indicates the increasing complexity of the technologies.

… how could systems‐guided metabolic engineering and synthetic biology contribute to sustainability goals?

One line of action is developing bio‐based alternatives to chemical processes that adopt biocatalysts developed through metabolic engineering, such as the production of degradable plastics, biofuels and both bulk and fine chemicals. These bio‐based alternatives are bound to capture a large portion of the current market as oil becomes more scarce and expensive. A second approach is to reduce and prevent emissions of harmful chemical waste at the point of manufacturing. Microorganisms naturally possess a considerable ability to degrade toxic molecules that has been substantially enhanced through directed evolution, genetic engineering or a combination of both. The resulting biocatalysts can be integrated in zero‐pollution industrial pipelines. By the same token, a number of CO_2_‐fixing microorganisms—not just cyanobacteria—and fermenters of highly complex municipal, commercial, sludge or agricultural biowaste can be genetically enhanced (or entirely reinvented) for superior performance to capture carbon in either mineral or organic forms, thereby allowing its conversion in value‐added products like sugars and polymers 6.

Increasing the range of biological organisms that can be predictably and reliably engineered […] is a critical next step towards fully realizing the potential of bio‐production.

Monitoring emissions of CO_2_ or other pollutants with biosensing devices based on the response of living cells to environmental pollutants has proven and will continue to be invaluable. Microorganisms are already being used to monitor pollutant levels, such as arsenic, in drinking water. When combined with adequate detection hardware, it is now possible to spot trinitrotoluene (TNT) residues in soil left by unexploded anti‐personnel mines with engineered bacteria 7. Furthermore, extensive bioremediation with biological agents, including phytoremediation, can be employed to remove pollutants from water or soil below tolerable concentrations.

Model‐guided design will become increasingly important as synthetic biology creates larger systems that interface gene circuits with other cellular systems…

The ongoing emission of greenhouse gases (CO_2_, CH_4_, N_2_O, chlorofluorocarbons and hydrofluorocarbons) and the overwhelming amount of microplastics in marine ecosystems are among the most pressing global challenges faced by our generation and require interventions that go beyond merely limiting emissions. The much‐debated geoengineering of planet Earth could potentially be complemented or even replaced by large‐scale bioremediation strategies to capture CO_2_ and to improve the capacity of marine microorganisms to degrade plastics in the environment. Whether or not the public will accept such unprecedented actions for handling emissions, which are reminiscent of *Terraforming* 8, remains to be seen (Fig 2). Indecisiveness in view of such threats has become one of the highest risks to developing a sustainable and lasting economy.

### Governance and regulation

This, in fact, raises the issue of how to properly regulate and employ these new technologies for both bioproduction and environmental management. Generally, governance should reflect common, broadly accepted values and build on existing bodies that command widespread support. In particular, we suggest two fundamental touchstones: The UN's Universal Declaration of Human Rights and the SDGs. The first states that all persons are entitled to basic rights and fundamental freedoms. As already discussed, the second are a call for action to meet the great challenges of our time. As new biotechnologies and their products emerge, a critical first step in evaluating these should include asking the following two questions: Is this technology in line with the Declaration of Human Rights? Does it advance the SDGs?

The much‐debated geoengineering of planet Earth could potentially be complemented or even replaced by large‐scale bioremediation strategies to capture CO_2_…

In seeking to reconcile conflicts and tensions between fundamental values—as well as regional or local values and preferences—the principles of proportionality and non‐discrimination can provide helpful guidance. Proportionality suggests for example that safety requirements are proportionate to a product's safety or risk profile and should not be disproportionately different to requirements for products with comparable or even identical safety profiles. A requirement to treat similar things similarly, and to treat things differently when there is a reasonable basis to do so, has been established as the non‐discrimination principle in many legal traditions 9.

The so‐called precautionary principle has gained much attention in policy discussions about the governance of new and emerging technologies, although there is considerable debate about its proper scope, content and application in particular cases, especially when food or the environment is at issue. According to the EU Commission, the precautionary principle requires that an intervention must be proportionate, non‐discriminatory and consistent, among other requirements, to achieve a balance between protecting society against unacceptable risk while not unnecessarily stifling innovation. Even where diverse views around the precautionary principle pertain, it is worth bearing in mind earlier considerations in the 1970s by the first generation of scientists working in the field of biotechnology who emphasized the need for robust regulation before moving into application, and the Organisation for Economic Co‐operation and Development's early guidance from the mid‐1980s to advance and assess step‐by‐step and case‐by‐case 10.

In seeking to reconcile conflicts and tensions between fundamental values […] the principles of proportionality and non‐discrimination can provide helpful guidance.

Given the enormous potential of biotechnology to help meet the SDGs and the controversy some areas of biotechnology have sparked, it is important to (re‐)establish a dialogue among all stakeholders to build and expand mutual understanding and a culture of trust between regulators, NGOs, scientists, industry and the public, while remaining respectful of established democratically legitimized administrative and judicial processes. Such discussion should take into account facts, feelings and value commitments, while seeking to obtain a clear view of risks and benefits. Only governance action that embraces such multi‐stakeholder discussions will be recognized as fair, unbiased, transparent and stable, which, ultimately, will benefit individuals, communities and society at large. Ensuring proper governance is essential for realizing biotechnology's contributions to achieving the UN's SDGs.

**Sidebar A: Further reading**

**On synthetic biology and metabolic engineering for the bio‐based production of chemicals, fuels and materials**

Choi SY, Park SJ, Kim WJ, Yang JE, Lee H, Shin J, Lee SY (2016) One‐step fermentative production of poly (lactate‐co‐glycolate) from carbohydrates in *Escherichia coli*. *Nat Biotechnol* 34: 435–440

Choi YJ, Lee SY (2013) Microbial production of short‐chain alkanes. *Nature* 502: 571–574

Galanie S, Thodey K, Trenchard IJ, Interrante MF, Smolke CD (2015) Complete biosynthesis of opioids in yeast. *Science* 349: 1095–1100

Luo ZW, Lee SY (2017) Biotransformation of *p*‐xylene into terephthalic acid by engineered *Escherichia coli. Nat Commun* 8: 15689

Paddon CJ, Westfall PJ, Pitera DJ, Benjamin K, Fisher K, McPhee D, Leavell MD, Tai A, Main A, Eng D *et al* (2013) High‐level semi‐synthetic production of the potent antimalarial artemisinin. *Nature* 496: 528–532

Qiao K, Wasylenko, TM, Zhou K, Xu P, Stephanopoulos G (2017) Lipid production in *Yarrowia lipolytica* is maximized by engineering cytosolic redox metabolism. *Nat Biotechnol* 35: 173–177

**On biotechnology for environmental sustainability and bioremediation**

Hicks N, Vik U, Taylor P, Ladoukakis E, Park J, Kolisis F, Jakobsen KS (2017) Using prokaryotes for carbon capture storage. *Trends Biotechnol* 35: 22–32

Logan BE, Rabaey K (2012) Conversion of wastes into bioelectricity and chemicals by using microbial electrochemical technologies. *Science* 337: 686–690

Lorenzo V, Marlière P, Solé R (2016) Bioremediation at a global scale: from the test tube to planet Earth. *Microb Biotechnol* 9: 618–625

Pieper DH, Reineke W (2000) Engineering bacteria for bioremediation. *Curr Opin Biotechnol* 11: 262–270

Schmidt M, de Lorenzo V (2016) Synthetic bugs on the loose: containment options for deeply engineered (micro) organisms. *Curr Opin Biotechnol* 38: 90–96

## Conflict of interest

The authors declare that they have no conflict of interest.

## References

[1] United Nations [UN] (2015) UN Sustainable development goals. Available at: http://www.un.org/sustainabledevelopment/sustainable-development-goals

[2] Schwab K (2016) The fourth industrial revolution. Geneva, Switzerland: World Economic Forum

[3] Lee JW , Na D , Park JM , Lee J , Choi S , Lee SY (2012) Systems metabolic engineering of microorganisms for natural and non‐natural chemicals. Nat Chem Biol 8: 536–546 22596205 10.1038/nchembio.970

[4] Nikel PI , Martínez‐García E , de Lorenzo V (2014) Biotechnological domestication of pseudomonads using synthetic biology. Nat Rev Microbiol 12: 368–379 24736795 10.1038/nrmicro3253

[5] Lee SY , Kim HU (2015) Systems strategies for developing industrial microbial strains. Nat Biotechnol 33: 1061–1072 26448090 10.1038/nbt.3365

[6] Antonovsky N , Gleizer S , Noor E , Zohar Y , Herz E , Barenholz U , Zelcbuch L , Amram S , Wides A , Tepper N *et al* (2016) Sugar synthesis from CO2 in *Escherichia coli* . Cell 166: 115–125 27345370 10.1016/j.cell.2016.05.064 PMC4930481

[7] Belkin S , Yagur‐Kroll S , Kabessa Y , Korouma V , Septon T , Anati Y , Zohar‐Perez C , Rabinovitz Z , Nussinovitch A , Agranat AJ (2017) Remote detection of buried landmines using a bacterial sensor. Nat Biotechnol 35: 308–310 28398330 10.1038/nbt.3791

[8] Solé S , Montañez R , Duran‐Nebreda S (2015) Synthetic circuit designs for earth terraformation. Biol Direct 10: 37 26187273 10.1186/s13062-015-0064-7 PMC4506446

[9] United Nations [UN] (2015) Equality and non‐discrimination. Available at: https://www.un.org/ruleoflaw/thematic-areas/human-rights/equality-and-non-discrimination/

[10] The Organization for Economic Co‐operation and Development (OECD) (1986) Recombinant DNA safety considerations. Paris, France: OECD Council

